# Rhinitis, sinusitis and ocular disease – 2039. Effect of intranasal steroid on cough symptom in patients with upper airway couth syndrome

**DOI:** 10.1186/1939-4551-6-S1-P171

**Published:** 2013-04-23

**Authors:** Jeong-Eun Kim, Jun-Hwi Song, Kyeung-Woo Kang

**Affiliations:** 1 Department of Internal Medicine, Samsung Changwon Hospital, Sungkyunkwan University School of Medicine, Changwon, South Korea

## Background

Upper airway cough syndrome (UACS) with rhinosinusitis is a major cause of chronic cough. Little is, however, known about the effects of medications used for chronic rhinosinusitis on symptom improvement in patients with chronic cough. The objective of this study is to observe outcome difference according to the medications used in patients with chronic cough caused by UACS.

## Methods

Medical records of patients, who visited our clinic for chronic cough caused by UACS from Jan 2011 to May 2012, were reviewed retrospectively. Patients with other diseases including bronchial asthma, gastroesophageal reflux disorder, and eosinophilic bronchitis were excluded. The medications and the results of paranasal sinus (PNS) series at 1^st^ visit were evaluated. According to the improvement of cough at 2^nd^ visit, the patients were divided into improved (no or a few cough) and not improved (no or a little improvement) groups. The effects of medications on cough were analyzed by multivariate logistic regression.

## Results

The medications at 1^st^ visit were composed of antihistamines, decongestants, antibiotics, leukotriene antagonists, and intranasal steroids. All the patients were treated with antitussives. The number of patients who used intranasal steroids was higher in improved group than in not improved group. Intranasal steroids were the only medication to improve cough significantly (OR 3.4, 95% CI [1.1-10.2], *p*=0.033), which was more significant in patients with chronic rhinosinusitis finding on PNS (OR 15.7, 95% CI [2.0-124.7], *p*=0.009).

## Conclusions

Intranasal steroid use may be beneficial to improve cough symptom in patients with UACS, in particular those with chronic rhinosinusitis finding on PNS.

